# Complete genome sequence of *Rhizobium leguminosarum* bv. *trifolii* strain WSM1325, an effective microsymbiont of annual Mediterranean clovers.

**DOI:** 10.4056/sigs.852027

**Published:** 2010-06-15

**Authors:** Wayne Reeve, Graham O’Hara, Patrick Chain, Julie Ardley, Lambert Bräu, Kemanthi Nandesena, Ravi Tiwari, Alex Copeland, Matt Nolan, Cliff Han, Thomas Brettin, Miriam Land, Galina Ovchinikova, Natalia Ivanova, Konstantinos Mavromatis, Victor Markowitz, Nikos Kyrpides, Vanessa Melino, Matthew Denton, Ron Yates, John Howieson

**Affiliations:** 1 Centre for *Rhizobium* Studies, Murdoch University, Western Australia, Australia; 2 DOE Joint Genome Institute, Walnut Creek, California, USA; 3 Lawrence Livermore National Laboratory, Livermore, California, USA; 4 Los Alamos National Laboratory, Bioscience Division, Los Alamos, New Mexico, USA; 5 Oak Ridge National Laboratory, Oak Ridge, Tennessee, USA; 6 Biological Data Management and Technology Center, Lawrence Berkeley National Laboratory, Berkeley, California, USA; 7 Department of Primary Industries, Victoria, Australia; 8 Department of Agriculture and Food, Western Australia, Australia

**Keywords:** microsymbiont, non-pathogenic, aerobic, Gram-negative rod, root-nodule bacteria, nitrogen fixation, *Alphaproteobacteria*

## Abstract

*Rhizobium leguminosarum* bv *trifolii* is a soil-inhabiting bacterium that has the capacity to be an effective nitrogen fixing microsymbiont of a diverse range of annual *Trifolium* (clover) species. Strain WSM1325 is an aerobic, motile, non-spore forming, Gram-negative rod isolated from root nodules collected in 1993 from the Greek Island of Serifos. WSM1325 is produced commercially in Australia as an inoculant for a broad range of annual clovers of Mediterranean origin due to its superior attributes of saprophytic competence, nitrogen fixation and acid-tolerance. Here we describe the basic features of this organism, together with the complete genome sequence, and annotation. This is the first completed genome sequence for a microsymbiont of annual clovers. We reveal that its genome size is 7,418,122 bp encoding 7,232 protein-coding genes and 61 RNA-only encoding genes. This multipartite genome contains 6 distinct replicons; a chromosome of size 4,767,043 bp and 5 plasmids of size 828,924 bp, 660,973 bp, 516,088 bp, 350,312 bp and 294,782 bp.

## Introduction

The productivity of agricultural systems is heavily dependent on nitrogen (N) [1]. The requirement for N-input can be met by the application of exogenous N-fertilizer manufactured through the Haber-Bosch process, but as the cost of fossil fuel-derived energy increases, so does the cost to manufacture and apply such fertilizer. Furthermore, there are inherent issues with the synthesis and application of N-fertilizer, including greenhouse gas emissions and run-off causing eutrophication. Alternatively, N can be obtained from symbiotic nitrogen fixation (SNF) by root nodule bacteria (rhizobia) on nodulated legumes [2]; this is a key biological process in natural and agricultural environments driven by solar radiation and utilizing atmospheric CO_2_. The commonly accepted figure for global SNF in agriculture is 50-70 million metric tons annually, worth in excess of U.S. $10 billion [3]. Rhizobia are applied across 400 million ha of agricultural land per annum to improve legume forage and crop production through symbiotic N-fixation [3].

The clover (*Trifolium*) nodulating *Rhizobium*  *R. leguminosarum* bv. *trifolii* is amongst the most exploited species of root-nodule bacteria in world agriculture. Clovers are widely grown pasture legumes and include both annual species (e.g. *T. subterraneum*) and perennial species (e.g. *T. pratense, T. repens* and *T. polymorphum*). Clovers are adapted to a wide range of environments, from sub-tropical to moist Mediterranean systems, and thus are important nitrogen-fixing legumes in many natural and agricultural regions of North and South America, Europe, Africa and Australasia [4].

*Rhizobium leguminosarum* bv. *trifolii* strain WSM1325 was isolated from a nodule recovered from the roots of an annual clover plant growing near Livadi beach on the Greek Cyclades island of Serifos in 1993 [5]. Strain WSM1325 is of particular interest because it is a highly effective nitrogen-fixing microsymbiont of a broad range of annual clovers of Mediterranean origin [5] and is also saprophytically competent in acid, infertile soils of both Uruguay and southern Australia [6]. Strain WSM1325 is an effective microsymbiont under competitive conditions for nodulation in what appears to be a host-mediated selection process [7].

As well as being a highly effective inoculant strain for annual *Trifolium* spp., strain WSM1325 is compatible with key perennial clovers of Mediterranean origin used in farming, such as *T. repens* and *T. fragiferum,* and is therefore one of the most important clover inoculants used in agriculture. However, WSM1325 is incompatible with American and African clovers, sometimes nodulating but never fixing N [5]. This is in contrast to other *Rhizobium leguminosarum* bv. *trifolii* strains, such as WSM2304, which are effective at N-fixation with some perennial American clovers, but ineffective with the Mediterranean clovers [5-7].

Here we present a summary classification and a set of features for *R. leguminosarum* bv. *trifolii* strain WSM1325 (Table 1), together with the description of a complete genome sequence and annotation.

**Table 1:** Classification and general features of *R. leguminosarum* bv. *trifolii* WSM1325 according to the MIGS recommendations [8].

**MIGS ID**	**Property**	**Term**	**Evidence code**
	Current classification	Domain *Bacteria*	TAS [9]
		Phylum *Proteobacteria*	TAS [10,11]
		Class *Alphaproteobacteria*	TAS [12,13]
		Order *Rhizobiales*	TAS [13,14]
		Family *Rhizobiaceae*	TAS [15,16]
		Genus *Rhizobium*	TAS [17-19]
		Species *Rhizobium leguminosarum*	TAS [18,20]
		Biovar *trifolii* Strain WSM1325	
	Gram stain	negative	TAS [17]
	Cell shape	rod	TAS [17]
	Motility	motile	TAS [17]
	Sporulation	non-sporulating	TAS [17]
	Temperature range	mesophile	TAS [17]
	Optimum temperature	28°C	TAS [17]
	Salinity	unknown	TAS [17]
MIGS-22	Oxygen requirement	aerobic	TAS [17]
	Carbon source	glucose, mannitol, glutamate	TAS [5-7]
	Energy source	chemoorganotroph	
MIGS-6	Habitat	Soil, root nodule, host	TAS [5-7]
MIGS-15	Biotic relationship	Free living, Symbiotic	TAS [5-7]
MIGS-14	Pathogenicity	none	NAS [17]
	Biosafety level	1	TAS [21]
	Isolation	Root nodule	TAS [22]
MIGS-4	Geographic location	Livadi beach, Serifos, Cyclades, Greece	TAS [22]
MIGS-5	Nodule collection date	April 1993	TAS [22]
MIGS-4.1 MIGS-4.2	Longitude Latitude	24.518901 37.147034	TAS [22]
MIGS-4.3	Depth	not reported	NAS
MIGS-4.4	Altitude	2m	TAS [22]

Evidence codes - TAS: Traceable Author Statement (i.e., a direct report exists in the literature); NAS: Non-traceable Author Statement (i.e., not directly observed for the living, isolated sample, but based on a generally accepted property for the species, or anecdotal evidence). These evidence codes are from http://www.geneontology.org/GO.evidence.shtml of the Gene Ontology project [23]. If the evidence code is IDA, then the property was directly observed by one of the authors or an expert mentioned in the acknowledgements.

## Classification and features

**Figure 1:**
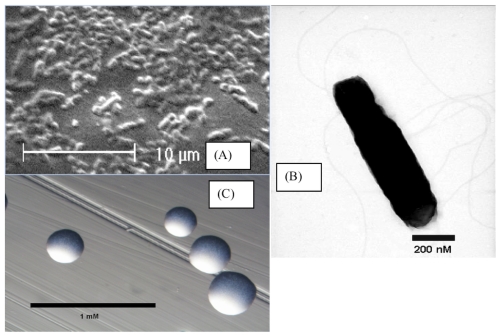
Images of *R. leguminosarum* bv. *trifolii* strain WSM1325 using scanning (A) and transmission (B) electron microscopy and the appearance of colony morphology on solid media (C).

*R. leguminosarum* bv. *trifolii* WSM1325 is a motile, Gram-negative, non-spore-forming rod (Figure 1A,B) in the *Rhizobiaceae* family of the class *Alphaproteobacteria* that forms mucoid colonies (Figure 1C) on solid media [24]. It has a mean generation time of 3.9 h in rich medium at the optimal growth temperature of 28°C [7].

**Figure 2:**
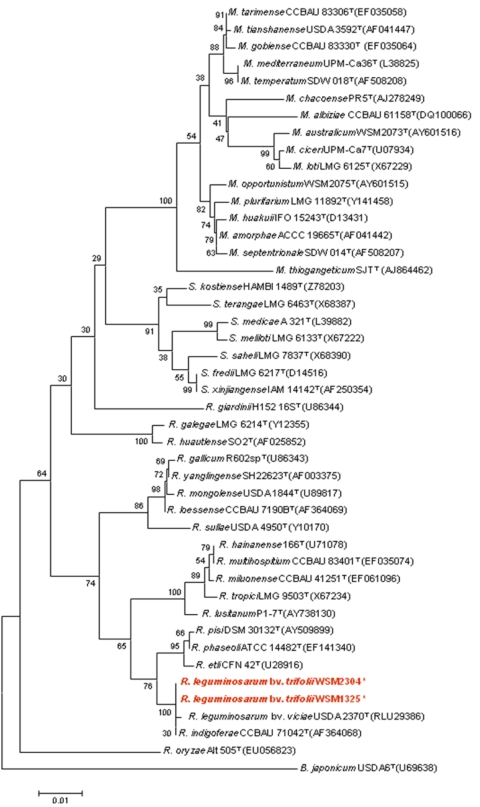
Phylogenetic tree showing the relationships of *R. leguminosarum* bv *trifolii* strain WSM1325 with the type strains of *Rhizobiaceae* based on aligned sequences of the 16S rRNA gene (1,440 bp internal region). All sites were informative and there were no gap-containing sites. Phylogenetic analyses were performed using MEGA, version 3.1 [25]. Kimura two-parameter distances were derived from the aligned sequences [26] and a bootstrap analysis [27] as performed with 500 replicates in order to construct a consensus unrooted tree using the neighbor-joining method [28] for each gene alignment separately. B.-*Bradyrhizobium*; M.-*Mesorhizobium*; R.-*Rhizobium*; S-*Ensifer* (*Sinorhizobium*). Type strains are indicated with a superscript T. Strains with a genome sequencing project registered in GOLD [22] are in bold red print. Published genomes are designated with an asterisk.

Figure 2 shows the phylogenetic neighborhood of *R. leguminosarum* bv. *trifolii* strain WSM1325 in a 16S rRNA-based tree. An intragenic fragment of 1,440 bp was chosen since the 16S rRNA gene has not been completely sequenced in many type strains. A comparison of the entire 16S rRNA gene of WSM1325 to completely sequenced 16S rRNA genes of other rhizobia revealed 100% gene sequence identity to the same gene of *R. leguminosarum* bv. *trifolii* strain WSM2304 but revealed a 1 bp difference to the same gene of *R. leguminosarum* bv. *viciae* strain 3841.

### Symbiotaxonomy

**Figure 3:**
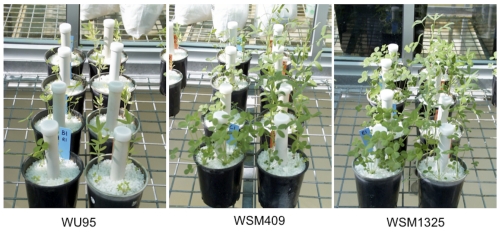
An illustration of the N-fixing capacity of *R. leguminosarum* bv. *trifolii* WSM1325 with four annual *Trifolium* spp. (*T. vesiculosum*, *T. dasyurum* (pots with orange tags), *T. isthmocarpum* and *T. spumosum* (pots with blue tags) in four replicates, front to back), compared with superseded Australian inoculants; far left WU95 (1968 to 1994), middle WSM409 (1994 to 2004) and right, WSM1325 (Australian commercial inoculant strain 2004 to present [29]).

*R. leguminosarum* bv. *trifolii* WSM1325 nodulates (Nod^+^) and fixes nitrogen effectively (Fix^+^) with a wide range of annual clovers of Mediterranean origin which are in commercial agriculture, globally. Examples of these clover species include *T. subterraneum*, *T. vesiculosum*, *T. purpureum T. glanduliferum, T. resupinatum, T. michellianum* and *T. incarnatum*. An illustration of the ability of WSM1325 to fix nitrogen effectively across a range of annual clover species is displayed in Figure 3. Additionally, WSM1325 is Fix^+^ with some Mediterranean perennial clovers such as *T. repens* and *T. fragiferum,* but is inconsistently Nod^+^, and consistently Fix^-^ with clovers of African and American origin [5,30]. Under conditions of competitive nodulation, WSM1325 may preferentially nodulate *T. purpureum* even when outnumbered 100:1 by WSM2304 [7].

## Genome sequencing and annotation information

### Genome project history

This organism was selected for sequencing on the basis of its environmental and agricultural relevance to issues in global carbon cycling, alternative energy production, and biogeochemical importance, and is part of the Community Sequencing Program at the US Department of Energy Joint Genome Institute (JGI) for projects of relevance to agency missions. The genome project is deposited in the Genomes OnLine Database [22] and the complete genome sequence in GenBank. Sequencing, finishing and annotation were performed by the DOE Joint Genome Institute (JGI). A summary of the project information is shown in Table 2.

**Table 2:** Genome sequencing project information for *R. leguminosarum* bv *trifolii* WSM1325.

**MIGS ID**	**Property**	**Term**
MIGS-31	Finishing quality	Finished
MIGS-28	Libraries used	Four genomic libraries: three Sanger libraries - 2 kb pTH1522, 8 kb pMCL200 and fosmid pcc1Fos and one 454 pyrosequence standard library
MIGS-29	Sequencing platforms	ABI3730xl, 454 GS FLX
MIGS-31.2	Sequencing coverage	16× Sanger; 20× pyrosequence
MIGS-30	Assemblers	Newbler version 1.1.02.15, phrap
MIGS-32	Gene calling method	Prodigal
	Genbank ID	CP001622 (Chomosome) ^a^ CP001623 (pR132501) ^b^ CP001624 (pR132502) ^c^ CP001625 (pR132503) ^d^ CP001626 (pR132504) ^e^ CP001627 (pR132505) ^f^
	Genbank Date of Release	May 7, 2009
	GOLD ID	Gc01039 ^g^
	NCBI project ID	20097
	Database: IMG	641736174 (draft) ^h^
	Project relevance	Symbiotic nitrogen fixation, agriculture

^a^ http://www.ncbi.nlm.nih.gov/nuccore/240856645; ^b^ http://www.ncbi.nlm.nih.gov/nuccore/240861211

^c^ http://www.ncbi.nlm.nih.gov/nuccore/240861949; ^d^ http://www.ncbi.nlm.nih.gov/nuccore/240862574

^e^ http://www.ncbi.nlm.nih.gov/nuccore/240863069; ^f^http://www.ncbi.nlm.nih.gov/nuccore/240863376

^g^ http://genomesonline.org/GOLD_CARDS/Gc01039.html

^h^ http://img.jgi.doe.gov/cgi-bin/pub/main.cgi?section=TaxonDetail&page=taxonDetail&taxon_oid=641736174

### Growth conditions and DNA isolation

*R. leguminosarum* bv. *trifolii* WSM1325 was grown to mid logarithmic phase in TY medium (a rich medium) [31] on a gyratory shaker at 28°C. DNA was isolated from 60 mL of cells using a CTAB (Cetyl trimethylammonium bromide) bacterial genomic DNA isolation method (http://my.jgi.doe.gov/general/index.html).

### Genome sequencing and assembly

The genome was sequenced using a combination of Sanger and 454 sequencing platforms. All general aspects of library construction and sequencing performed at the JGI can be found at http://www.jgi.doe.gov/. 454 Pyrosequencing reads were assembled using the Newbler assembler, version 1.1.02.15 (Roche). Large Newbler contigs were broken into 6,084 overlapping fragments of 1,000 bp and entered into assembly as pseudo-reads. The sequences were assigned quality scores based on Newbler consensus q-scores with modifications to account for overlap redundancy and to adjust inflated q-scores. A hybrid 454/Sanger assembly was made using the parallel phrap assembler (High Performance Software, LLC). Possible mis-assemblies were corrected with Dupfinisher or transposon bombing of bridging clones [32]. Gaps between contigs were closed by editing in Consed, custom primer walk or PCR amplification. A total of 2,155 Sanger finishing reads were produced to close gaps, to resolve repetitive regions, and to raise the quality of the finished sequence. Together, all sequence types provided 36× coverage of the genome. The error rate of the completed genome sequence is less than 1 in 100,000.

### Genome annotation

Genes were identified using Prodigal [33] as part of the Oak Ridge National Laboratory genome annotation pipeline, followed by a round of manual curation using the JGI GenePrimp pipeline [34]. The predicted CDSs were translated and used to search the National Center for Biotechnology Information (NCBI) nonredundant database, UniProt, TIGRFam, Pfam, PRIAM, KEGG, COG, and InterPro databases. Additional gene prediction analyses and functional annotation were performed within the Integrated Microbial Genomes (IMG-ER) platform (http://img.jgi.doe.gov/er) [35].

## Genome properties

**Figure 4:**
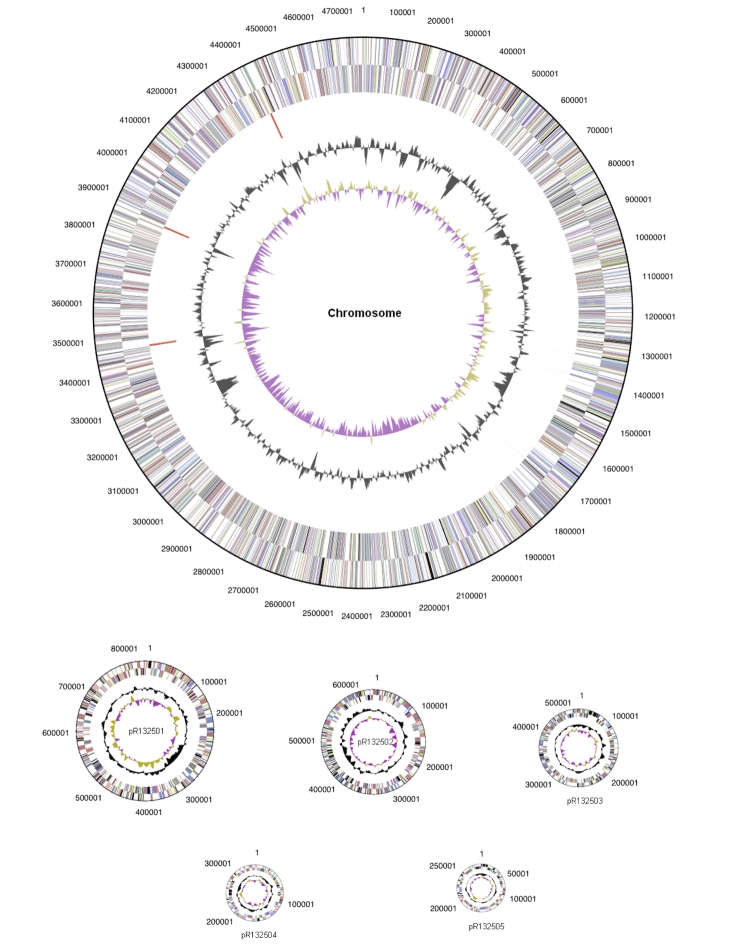
Graphical circular maps of the chromosome and plasmids of *R. leguminosarum* bv *trifolii* WSM1325. From outside to the center: Genes on forward strand (color by COG categories as denoted by the IMG platform), Genes on reverse strand (color by COG categories), RNA genes (tRNAs green, sRNAs red, other RNAs black), GC content, GC skew. Chromosome and plasmids are not drawn to scale.

The genome is 7,418,122 bp long with a 60.77% GC content (Table 3) and comprised of 6 replicons; one circular chromosome of size 4,767,043 bp and five circular plasmids of size 828,924 bp, 660,973 bp, 516,088 bp, 350,312 bp and 294,782 bp (Figure 4). Of the 7293 genes predicted, 7,232 were protein coding genes, and 61 RNA only encoding genes. Two hundred and thirty one pseudogenes were also identified. The majority of genes (74.21%) were assigned a putative function whilst the remaining genes were annotated as hypothetical proteins. The distribution of genes into COGs functional categories is presented in Table 4.

**Table 3:** Genome Statistics for *R. leguminosarum* bv *trifolii* WSM1325.

**Attribute**	**Value**	**% of Total**
Genome size (bp)	7,418,122	100.00
DNA coding region (bp)	6,485,014	87.42
DNA G+C content (bp)	4,507,991	60.77
Number of replicons	6	
Extrachromosomal elements	5	
Total genes	7,293	100.00
RNA genes	61	0.84
rRNA operons	3	
Protein-coding genes	7,232	99.16
Pseudo genes	231	3.17
Genes with function prediction	5,412	74.21
Genes in paralog clusters	1,947	26.70
Genes assigned to COGs	5,453	74.77
Genes assigned Pfam domains	5,497	75.37
Genes with signal peptides	1,554	21.31
Genes with transmembrane helices	1,629	22.34
CRISPR repeats	0	

**Table 4:** The number of predicted protein-coding genes of *R. leguminosarum* bv *trifolii* WSM1325 associated with the 21 general COG functional categories.

**Code**	**value**	**% age**	**Description**
J	195	2.70	Translation, ribosomal structure and biogenesis
A	1	0.01	RNA processing and modification
K	610	8.43	Transcription
L	180	2.49	Replication, recombination and repair
B	2	0.03	Chromatin structure and dynamics
D	35	0.48	Cell cycle control, mitosis and meiosis
Y	0	0.00	Nuclear structure
V	69	0.95	Defense mechanisms
T	358	4.95	Signal transduction mechanisms
M	323	4.47	Cell wall/membrane biogenesis
N	89	1.23	Cell motility
Z	1	0.01	Cytoskeleton
W	0	0.00	Extracellular structures
U	83	1.15	Intracellular trafficking and secretion
O	191	2.64	Posttranslational modification, protein turnover, chaperones
C	316	4.37	Energy production and conversion
G	688	9.51	Carbohydrate transport and metabolism
E	644	8.90	Amino acid transport and metabolism
F	108	1.49	Nucleotide transport and metabolism
H	190	2.63	Coenzyme transport and metabolism
I	227	3.14	Lipid transport and metabolism
P	322	4.45	Inorganic ion transport and metabolism
Q	160	2.21	Secondary metabolites biosynthesis, transport and catabolism
R	777	10.74	General function prediction only
S	586	8.10	Function unknown
-	1,779	24.60	Not in COGs
